# Field vs. Emergency Department Intubation: A Retrospective Review of Hospital Outcomes of Trauma Patients

**DOI:** 10.5811/westjem.41184

**Published:** 2025-05-19

**Authors:** Mitchell Vorce, Sagar Galwankar, Jarrod Shuck, Amit Agrawal

**Affiliations:** * Florida State College of Medicine, Department of Emergency Medicine Residency, Sarasota, Florida; † All India Institute of Medical Sciences, Department of Neurosurgery, Bhopal, Madhya Pradesh, India

## Abstract

**Introduction:**

Definitive airway management is crucial for severely injured trauma patients when basic pre-hospital interventions fail to provide adequate oxygenation and ventilation. Endotracheal intubation by emergency medical service (EMS) personnel is often necessary before reaching the emergency department (ED). While some studies suggest that advanced airway protocols in the pre-hospital setting improve survival in patients with severe head injuries, others indicate potential complications and adverse outcomes associated with pre-hospital intubation. In this study we aimed to evaluate whether trauma patients who underwent intubation by EMS in the field experienced different hospital outcomes compared to those intubated by physicians in the ED. Specifically, it assessed the impact of pre-hospital intubation on the number of days requiring mechanical ventilation, intensive care unit length of stay (ICU LOS), and overall hospital LOS.

**Methods:**

We conducted a retrospective chart review at a single, level II trauma center from January 1, 2019–December 31, 2023, involving trauma patients requiring intubation. Patients were divided into two groups: 608 patients ED department (ED ETT). Primary outcomes included days on mechanical ventilation, while secondary outcomes included ICU and hospital LOS. An independent *t*-test was performed to compare the differences in mean ventilator days, ICU LOS, and hospital LOS between the two groups, accepting *P*-value of <0.10 as significant.

**Results:**

The study included 1,010 patients, with a mean age of 55.5 years in the ED group and 52.5 years in the pre-hospital group. No statistically significant differences were found in mean ventilator days (4.1 ± 4.6 days for the ED group and 4.1 ± 5.7 days for the pre-hospital group), ICU LOS (5.8 ± 6.1 days in the ED ETT group vs 5.6 ± 7.4 days in the pre-hospital ETT group), or overall hospital LOS (10.1 ± 13.6 days in the ED group vs 10.2 ± 17.5 days in the pre-hospital group).

**Conclusion:**

These findings indicate no significant differences in patient outcomes between those intubated pre-hospital and those intubated in the ED. Further research is needed to make modifications to airway management protocols in the pre-hospital setting.

## INTRODUCTION

Definitive airway placement is commonly required in severely injured trauma patients when basic pre-hospital airway interventions are inadequate or ineffective at providing oxygenation and ventilation. Endotracheal intubation by paramedics or emergency medical service (EMS) responders is often required prior to arriving at the emergency department (ED) for a variety of reasons. Implementation of advanced airway protocols in the pre-hospital setting has been associated with improved survival in patients with severe head injuries leading some to suggest benefits in broadening indications for intubation by pre-hospital personnel.^1^ However, given the challenge of the procedure itself and potential complications, an argument could be made that more conservative pre-hospital airway measures and intubation in the ED could be a safer alternative. There is also evidence that pre-hospital intubation can be associated with higher rates of adverse outcomes and that replacement of bag-valve-mask ventilation with endotracheal intubation in the pre-hospital setting does not result in improved survival or improved neurologic outcomes.^2^,^3^,^4^

There are several challenges associated with pre-hospital intubation that could theoretically lead to higher rates of complications and poor patient outcomes. These include environmental hazards, limited equipment, suboptimal patient positioning, variations in skill levels among first responders, and limited ability to manage complications. Some studies have reported endotracheal tube (ET) misplacement rates by EMS personnel from 9% to as high as 25%.^M^isplacement of an ET tube carries significant risk of iatrogenic injury, and lack of first-pass success is likely to result in worse patient outcomes by prolonging periods without adequate oxygenation. Although the reported overall success rates of pre-hospital intubation have improved over time, there is still a significant difference in first-pass success and overall intubation success rate between pre-hospital, non-physician personnel and physicians, which can result in prolonged transport times and a potential delay in receiving definitive care in the ED.^6^,^7^ For this reason, advanced airway management by non-physicians remain somewhat controversial.

There are several advantages to performing endotracheal intubation in the ED including availability of adjunctive medications, advanced monitoring equipment, additional support staff, and airway specialist oversight, among others. Multiple studies have reported increased mortality when patients were intubated in the pre-hospital setting.^8^,^9^,^10^ There is a considerable amount of conflicting data regarding the outcomes of patients intubated in the pre-hospital setting compared to those intubated in the ED. Most of the existing literature comparing outcomes between these two patient populations analyze mortality rate and neurologic function as the primary outcomes. Few studies have compared the differences in number of days requiring mechanical ventilation, intensive care unit length of stay (ICU LOS), and hospital LOS. Understanding these additional metrics is important since they can significantly impact overall healthcare costs and resource utilization. Additional research into these outcomes may provide a more comprehensive understanding of the implications of pre-hospital vs in-hospital intubation.

## OBJECTIVES

We conducted this study to understand whether the hospital course of trauma patients requiring intubation differs if the intubation is performed by EMS personnel in the field vs by physicians in the ED. Identification of worse or improved outcomes could potentially lead to modification of existing airway intervention protocols for EMS. In this study we aied to evaluate the impact of pre-hospital intubation on the number of days requiring mechanical ventilation, ICU LOS, and hospital LOS.

**Population Health Research Capsule**

What do we already know about this issue?

*It is unknown whether performing intubation of trauma patients in the pre-hospital setting vs. in the emergency department leads to better outcomes.*

What was the research question?

*Is there a difference between patient outcomes for trauma patients intubated pre-hospital vs. in the ED? Does the location of intubation affect the hospital course of trauma patients?*

What was the major finding of the study?

*We found no difference between pre-hospital and in-hospital intubation groups by ventilator days (mean 4.1 days for each, p = 0.87), intensive care unit length of stay (LOS) (mean 5.8 vs. 5.6 days, p = 0.62), and hospital LOS (10.1 vs. 10.2 days, p = 0.98).*

How does this improve population health?

*Pre-hospital and in-hospital intubation showed similar critical care outcomes for trauma patients, supporting pre-hospital intubation by Emergency Medical Services. This is important, particularly in rural areas with long transport times.*

## METHODS

We adhered to several criteria proposed by Worster et al to ensure optimal retrospective chart review and study. These include using trained abstractors, finding clear inclusion and exclusion criteria, using clearly defined variables, discussing interobserver reliability, identifying and abstracting from one health record database, performing simple random sampling, agreeing upon management of missing data, and receiving institutional review board approval.^11^ The data from this retrospective chart review was collected from health records from Sarasota Memorial Hospital Trauma Department from January1, 2019– December 31, 2023. This is a level II trauma center in Sarasota, FL. The patients were transported to this hospital by several EMS agencies including Sarasota County, Manatee County, Bayfront Medical Center, Tampa General Healthcare System, and North Port Fire Rescue, with the vast majority being Sarasota County EMS services. The study population was trauma patients who were brought to the ED and required endotracheal intubation. The patients were divided into two groups, group 1—patients intubated in a pre-hospital setting (pre-hospital ETT); and group 2—patients intubated in the (ED ETT).

The primary outcome measures included the number of days requiring mechanical ventilation (with one ventilator day being counted if the patient remained intubated at any point within a 24-hour period). Secondary outcome measures consisted of LOS in the ICU and the overall hospital LOS. The inclusion criteria were trauma patients requiring intubation, intubation occurring prior to arrival or while in the ED, and patients ≥18 years of age. The exclusion criteria were patients who expired pre-hospital or while in the ED, and the patients who were intubated after being transferred out of the ED. The details retrieved were age, sex, any comorbidities (alcoholism, substance use disorder, chronic obstructive pulmonary disease (COPD), smoker, congestive heart failure (CHF), renal failure, and dementia); whether the patients had pre-hospital intubation and ED intubation; number of ventilator days; ICU LOS; hospital LOS, Injury Severity Score (ISS), mechanism of injury (MOI), ED or hospital disposition (ED Dispo or Hospital DC Dispo), transport method (air/ground/private) and transport agency (EMS organization).

The original dataset included patients who were intubated in the pre-hospital setting using a supraglottic airway device (not a definitive endotracheal airway) and subsequently had the supraglottic device removed and replaced by an endotracheal tube in the ED. These patients were listed in both the pre-hospital ETT and ED ETT groups in the original dataset. These patients were considered to be intubated in the ED. They were removed from the pre-hospital ETT group and remained in the ED ETT group. This decreased the pr-hospital ETT total from 672 to 608 patients. The injuries were categorized into various MOI by using keyword search from the *International Classification of Diseases*, 10^th^ Rev (ICD-10) E-code Injury Descriptor Text provided. An independent *t*-test was performed to determine whether there was any statistically significant difference between mean ventilator days, ICU LOS, and hospital LOS of the two groups, accepting *P*-value of <0.05 as significant.

## RESULTS

In the study there were a total 1,010 patients: pre-hospital, l,608; and ED, 402).

### Descriptive Statistics (Table 1)

The mean age in the ED ETT group was 55.5 ± 21 years (range 18 to 96 years), while in the Pre-hospital ETT group, it was 52.5 ± 20.5 years (range 18 to 103 years). The mean Injury Severity Score (ISS) was 17.8 ± 13.4 in the ED ETT group (range 1 to 75) and 20 ± 15.4 in the Pre-hospital ETT group (range 1 to 75). The ED ETT group had a mean of 4.1 ± 4.6 ventilator days (range 0 to 40), and the Pre-hospital ETT group had a mean of 4.1 ± 5.7 ventilator days (range 0 to 78). ICU length of stay (LOS) was 5.8 ± 6.1 days for the ED ETT group (range 0 to 48) and 5.6 ± 7.4 days for the Pre-hospital ETT group (range 0 to 87). The hospital length of stay (LOS) was 10.1 ± 13.6 days for the ED ETT group (range 0 to 129) and 10.2 ± 17.5 days for the Pre-hospital ETT group (range 0 to 147). At the time of intubation, Glasgow Coma Scale (GCS) scores were 10.7 ± 4.7 for the ED ETT group (range 3 to 15) and 4.4 ± 2.6 for the Pre-hospital ETT group (range 3 to 15). There is a difference in the total count of patients reported in age and ISS categories. This is due to a small number of patients in each group not having an age or ISS included in the data set and is not expected to significantly affect the primary outcomes examined.

### Age Distribution

The majority of the patients were young adults, particularly in the 19–30 age group, with 58 cases in ED ETT group and 101 in Pre-hospital ETT group. This was followed by 31–40 age group 53 cases in ED ETT and 95 in Pre-hospital ETT.

### Sex Distribution

In the ED ETT group, there were 128 (31.8%) females and 274 (68.2%) males. In the Pre-hospital ETT category, there were 165 (27.1%) females and 443 (72.9%) males.

### Mechanism of Injuries

Patients were categorized into various mechanisms of injury (MOI) by using keyword search from the ICD-10 E-code Injury Descriptor Text provided in the original dataset. The categories include motor vehicle collision (MVC), fall, assault, intentional self-harm, gunshot wound (GSW), burn, unspecifiedunintentional trauma, unspecified penetrating trauma, submersion injury, animal bite, and electric injury. The number of patients presenting for each type of injury was compared based on which group they were included in, those intubated in the emergency department (ED ETT) and those intubated in the pre-hospital setting by emergency medical services personnel (pre-hospital ETT). The number of each patient and percentage of the group that each category represents was included. In the ED ETT group, motor vehicle collisions (MVC) were the most prevalent mechanism of injuries 183 cases (52.0%) followed by falls 83 cases (23.6%) and assaults 32 cases. Similarly, in the pre-hospital ETT group, motor vehicle collisions (MVC) were the most common with 295 cases (53.2%) followed by falls 138 cases (24.9%) and assaults 32 cases (5.8%).

### Details of Co-Morbidities

Co-morbidities in each group, those intubated in the emergency department (ED ETT) and those intubated in the pre-hospital setting (Pre-hospital ETT), included tobacco smoking history (smoker), patients with a documented history of alcoholism (alcoholism), chronic obstructive pulmonary disease (COPD), documented history of substance abuse (substance abuse), congestive heart failure (CHF), dementia, and renal failure. In the Emergency Department (ED) ETT, the most common co-morbidities were smoker—64 cases (30.9%), followed by alcoholism with 46 cases (22.2%), and chronic obstructive pulmonary disease (COPD) with 29 cases (14.0%), respectively. Other co-morbidities were congestive heart failure (CHF) and substance abuse in 25 cases (12.1%) and 26 cases (12.6%), respectively. Dementia and renal failure were in 12 cases (5.8%) and 5 cases (2.4%), respectively. The Pre-hospital ETT group also had a higher prevalence of smokers with 91 cases (35.1%), followed by alcoholism with 62 cases (23.9%) and COPD with 27 cases (10.4%), respectively. Dementia and CHF were present in 16 cases (6.2%) and 24 cases (9.3%), respectively. Substance abuse was reported in 34 cases (13.1%), and renal failure was reported in 5 cases (1.9%).

### Correlation Analysis

The correlation matrices among clinical factors within each group are shown in Table-2. In the ED ETT group, Ventilator Days and ICU LOS were positively correlated (r = 0.831), indicating that patients needing prolonged ventilation also had extended ICU stays. Similarly, in the Pre-hospital ETT group, Ventilator Days correlated strongly with ICU LOS (r = 0.910), reflecting a pattern comparable with ED ETT group. Initial GCS was negatively correlated with ISS in both the ED ETT (r = −0.142) and Pre-hospital ETT groups (r = −0.216), suggesting that more severely injured patients presented with lower GCS scores. Mean values and standard deviations of the selected variables are shown in Table 3. ED ETT patients had a mean age of 55.41 years, compared to 52.53 years for pre-hospital ETT patients. Initial GCS scores were significantly different between the two settings (ED: 10.67 ± 4.68 vs. Pre-hospital: 4.41 ± 2.64). Age (t = 2.16, P < .05) and ISS (t = −2.23, P < .05) differed significantly. Ventilator Days (t = −0.15, P > .05), ICU LOS (t = 0.48, P > .05), and hospital LOS (t = −0.02, P > .05), did not show significant differences between the two groups. Initial GCS was notably different (t = 27.07, P < .05), with patients intubated pre-hospital having poorer GCS scores than those intubated in the ED.

## DISCUSSION

There are areas of debate regarding whether pre-hospital intubation is beneficial or whether intubation should be delayed and performed in the ED.^13^–^17^ Although guidelines exist,^18^,^19^ there is ongoing research and discussion to optimize protocols.^20^–^26^ Our goal in this study was to examine the outcomes of trauma patients who required endotracheal intubation, either in the pre-hospital setting or while in the ED. The goal ws to identify any significant differences in ICU LOS, and overall hospital LOS. Understanding these additional metrics is important since they can significantly impact overall healthcare costs and resource utilization.

These findings suggest that there were no significant differences in the outcomes examined. The lack of statistically significant differences implies that, in the study population, intubation in the pre-hospital setting did not worsen or improve patient outcomes when compared to patients who were intubated in the ED setting. Compared to patients who were intubated in the ED, those who were intubated in the pre-hospital setting were more severely injured, with higher ISS and lower GCS scores. Patients who were more severely injured and intubated in the pre-hospital setting, which presents several unique challenges, would be expected to have longer, more protracted hospital courses and poorer outcomes. Patients in the ED ETT group also had a significant difference in age, having a higher average age compared to the pre-hospital ETT group, which may make patients more likely to experience a longer, more complicated hospital course. Despite this, there were no notable differences between the two groups. The lack of a noticeable difference in hospital course may lend some evidence in support of pre-hospital intubation in severely injured trauma patients.

Many studies have questioned the utility of pre-hospital endotracheal intubation, and there are several that may even indicate a potential for worse outcomes after pre-hospital endotracheal intubation vs basic pre-hospital airway management.^6^, ^26^ There are higher rates of failed intubation when performed in the pre-hospital setting with a number of possible complications, and most studies show no improvement in neurologic outcomes or long-term survival rates.^1^,^5^,^8^ There is also the problem of intubation attempts detracting from other critical resuscitative efforts, such as high-quality chest compressions, defibrillation, medication administration, and non-invasive ventilation. Despite these challenges and the multiple studies calling into question the utility of pre-hospital intubation, there are a great deal of confounding variables in these studies. There is still a need for more evidence before more definitive conclusions can be made.

## LIMITATIONS

The retrospective nature of the study limits its broader applicability. Additionally, to increase the generalizability of the findings, data from a larger number of institutions and peripheral settings with varying complexities and geographical differences are needed in a prospective manner. This study design lacks external validity due to being conducted in a single center in a small, isolated geographic region and would not be generalizable to the larger population. It was performed retrospectively over a five-year period, which introduces the possibility of selection bias, mis-classification bias, information bias, observer bias, and recall bias. The study period did overlap with the COVID-19 pandemic. It is possible that some EMS agencies may have altered their airway management protocols to minimize exposure to EMS personnel. However, recommendations for pre-hospital airway management in patients with suspected COVID-19 infection focused primarily on proper personal protective equipment (PPE) use and utilization of high efficiency particle arresting filters following endotracheal intubation or supraglottic airway (SGA) placement.

Indications for advanced airway placement were not changed. Video assisted laryngoscopy (VL) was recommended over direct visualization, if available, and placement of a SGA was advised if VL was unavailable.^28^ This may have led to a larger number of patients who would have traditionally been intubated in the pre-hospital setting, being intubated in the ED. This could have resulted in more severely injured, critically ill patients being included in the ED ETT group; however, this outcome was not seen, suggesting the EMS agencies involved in this study did not significantly alter their protocols, focused on proper PPE use, or had access to VL. There are several additional considerations that need to be made when interpreting these findings.

Although the majority of the patients in the pre-hospital ETT group were treated by one EMS agency, there were several other EMS agencies included in the study. There is a great deal of variation in intubation success rates by EMS companies.^5^ We did not have access to the field airway management protocols, but this variation may be due to differences in airway assessment, management, and responder skill level among the various agencies. Level of EMS personnel training and experience is also variable. If the patient was brought to the hospital via ground transport, the paramedic or, in rare cases, the EMS physician, who is an emergency medicine resident or attending physician assigned to work with the crew, was the clinician performing the intubation. Patients transported by flight teams were intubated by flight paramedics, but the majority of patients in each group were transported by ground teams. Details of the pre-hospital management including the level of training for the personnel performing intubation and transport time, among others, were not obtainable. All these factors have potential to affect intubation success rate, hospital course, and patient outcome.

In addition to variations in pre-hospital personnel training, the experience level of those performing intubation in the ED also varied. At this institution, there is an emergency medicine residency. It is protocol for residents to perform the first intubation attempt, followed by the attending emergency physician, and finally by the anesthesia attending, if all other attempts are unsuccessful. There were several situations in which the number of ventilator days, length of ICU stay, and length of hospital stay were reported to be much less than one would expect. This includes patients who expired in the inpatient setting, patients who were transferred to an outside facility requiring higher level of care, patients who were discharged from the hospital to hospice care, and patients who left the hospital against medical advice.

## CONCLUSIONS

Currently, there is insufficient evidence to justify any changes to pre-hospital advanced airway management protocols. Additional research is needed in this area. We did not find any significant differences in terms of reliance on mechanical ventilation or length of hospital stay between the two groups. Given these findings, it is essential to conduct larger, multicenter studies to evaluate patient outcomes comprehensively and to explore additional factors that may influence the effectiveness of pre-hospital intubation. By doing so, we can better understand the nuances of airway management in trauma care and develop evidence-based guidelines that optimize patient safety and outcomes. Collaborative efforts between EMS responders and hospital systems will be vital in addressing these questions and improving overall care for trauma patients. Additional research into these outcomes may provide a more comprehensive understanding of the implications of pre-hospital versus in-hospital intubation.

## Figures and Tables

**Table 1:** The descriptive statistics of the following variables of the two groups, emergency department intubation group (ED ETT) and pre-hospital intubation group (Pre-hospital ETT), are displayed here. Age is displayed in years. Injury severity score (ISS) and Glasgow coma scale (GCS) are on a numerical scale. Ventilator days (vent days), intensive care unit length of stay (ICU LOS), and hospital length of stay (hospital LOS) are in number of days. The total number (count), mean, standard deviation (std), minimum (min), first quartile (25%), median (50%), third quartile (75%), and maximum (max) values for each variable are listed.

	Age		ISS		Ventilator Days		ICU LOS		Hospital LOS		Initial GCS	
					
	ED ETT	Pre-hospital ETT	ED ETT	Pre-hospital ETT	ED ETT	Pre-hospital ETT	ED ETT	Pre-hospital ETT	ED ETT	Pre-hospital ETT	ED ETT	Pre-hospital ETT
count	391	594	394	587	402	608	402	608	402	608	402	608
mean	55.5	52.5	17.8	20	4.1	4.1	5.8	5.6	10.1	10.2	10.7	4.4
std	21	20.5	13.4	15.4	4.6	5.7	6.1	7.4	13.6	17.5	4.7	2.6
min	18	18	1	1	0	0	0	0	0	0	3	3
25%	36	34	6.5	8	2	1	2	1	2	1	7	3
50%	58	53	17	19	2	2	4	3	6	4	13	3
75%	73	68	26	27	5	5	8	7	14	11	15	6
max	96	103	75	75	40	78	48	87	129	147	15	15

**Table 2:** R-values of correlation matrix for the following variables of the two groups, emergency department intubation group (ED ETT) and pre-hospital intubation group (Pre-hospital ETT) are listed in the table below. Variables of the two groups that were analyzed include: age, injury severity score (ISS), ventilator days (vent days), intensive care unit length of stay (ICU LOS), hospital length of stay (hospital LOS), and initial Glasgow coma scale score (initial GCS). Age is in years. ISS and GCS are on a numerical scale. Vent days, ICU LOS, and hospital LOS are in number of days. The total number (count), mean, standard deviation (std), minimum (min), first quartile (25%), median (50%), third quartile (75%), and maximum (max) values for each variable are listed.

Correlation Matrix for ED ETT						

	Age	ISS	Vent Days	ICU LOS	Hospital LOS	Initial GCS
Age	1	−0.0142	0.119555	0.154048	−0.05381	0.072941
ISS	−0.0142	1	0.266635	0.265233	0.158705	−0.14235
Vent days	0.119555	0.266635	1	0.830989	0.523683	0.023537
ICU LOS (days)	0.154048	0.265233	0.830989	1	0.655022	0.037941
Hospital LOS (days)	−0.05381	0.158705	0.523683	0.655022	1	0.015997
Initial GCS	0.072941	−0.14235	0.023537	0.037941	0.015997	1
Correlation Matrix for Pre-hospital ETT						
	**Age**	**ISS**	**Vent Days**	**ICU LOS**	**Hospital LOS**	**Initial GCS**
Age	1	−0.11417	0.018234	0.013062	−0.08269	0.066854
ISS	−0.11417	1	0.166451	0.16036	0.121373	−0.2159
Vent days	0.018234	0.166451	1	0.909962	0.628391	−0.04413
ICU LOS (days)	0.013062	0.16036	0.909962	1	0.731803	−0.0368
Hospital LOS (days)	−0.08269	0.121373	0.628391	0.731803	1	−0.04758
Initial GCS	0.066854	−0.2159	−0.04413	−0.0368	−0.04758	1

**Table 3:** T-Test results of the following variables of the two groups, emergency department intubation group (ED ETT) and pre-hospital intubation group (pre-hospital ETT) are listed in the table below. Variables of the two groups that were analyzed include: age, injury severity score (ISS), ventilator days (vent days), intensive care unit length of stay (ICU LOS), hospital length of stay (hospital LOS), and initial Glasgow coma scale score (initial GCS). Age is in years. ISS and GCS are on a numerical scale. Vent days, ICU LOS, and hospital LOS are in number of days

Variable	ED ETT (Mean ± SD)	Pre-hospital ETT (Mean ± SD)	t-statistic	*P*-value
Age	55.41 ± 20.97	52.53 ± 20.53	2.16	0.031
ISS	17.82 ± 13.30	19.90 ± 15.34	−2.23	0.026
Vent days	4.08 ± 4.59	4.13 ± 5.73	−0.15	0.88
ICU LOS (days)	5.82 ± 6.13	5.60 ± 7.44	0.48	0.63
Hospital LOS (days)	10.15 ± 13.56	10.17 ± 17.48	−0.02	0.98
Initial GCS	10.67 ± 4.68	4.41 ± 2.64	27.07	0
